# Analysis of time-dependent adaptations in whole-body energy balance in obesity induced by high-fat diet in rats

**DOI:** 10.1186/1476-511X-10-99

**Published:** 2011-06-16

**Authors:** Mandy So, Mandeep P Gaidhu, Babak Maghdoori, Rolando B Ceddia

**Affiliations:** 1 School of Kinesiology and Health Science - Muscle Health Research Center York University, Toronto, ON, Canada

**Keywords:** Energy efficiency, brown adipose tissue, UCP-1, energy expenditure, leptin, ACC, skeletal muscle, fat oxidation, food intake, adiposity

## Abstract

**Background:**

High-fat (HF) diet has been extensively used as a model to study metabolic disorders of human obesity in rodents. However, the adaptive whole-body metabolic responses that drive the development of obesity with chronically feeding a HF diet are not fully understood. Therefore, this study investigated the physiological mechanisms by which whole-body energy balance and substrate partitioning are adjusted in the course of HF diet-induced obesity.

**Methods:**

Male Wistar rats were fed *ad libitum *either a standard or a HF diet for 8 weeks. Food intake (FI) and body weight were monitored daily, while oxygen consumption, respiratory exchange ratio, physical activity, and energy expenditure (EE) were assessed weekly. At week 8, fat mass and lean body mass (LBM), fatty acid oxidation and uncoupling protein-1 (UCP-1) content in brown adipose tissue (BAT), as well as acetyl-CoA carboxylase (ACC) content in liver and epidydimal fat were measured.

**Results:**

Within 1 week of *ad libitum *HF diet, rats were able to spontaneously reduce FI to precisely match energy intake of control rats, indicating that alterations in dietary energy density were rapidly detected and FI was self-regulated accordingly. Oxygen consumption was higher in HF than controls throughout the study as whole-body fat oxidation also progressively increased. In HF rats, EE initially increased, but then reduced as dark cycle ambulatory activity reached values ~38% lower than controls. No differences in LBM were detected; however, epidydimal, inguinal, and retroperitoneal fat pads were 1.85-, 1.89-, and 2.54-fold larger in HF-fed than control rats, respectively. Plasma leptin was higher in HF rats than controls throughout the study, indicating the induction of leptin resistance by HF diet. At week 8, UCP-1 content and palmitate oxidation in BAT were 3.1- and 1.5-fold higher in HF rats than controls, respectively, while ACC content in liver and epididymal fat was markedly reduced.

**Conclusion:**

The thermogenic response induced by the HF diet was offset by increased energy efficiency and time-dependent reduction in physical activity, favoring fat accumulation. These adaptations were mainly driven by the nutrient composition of the diet, since control and HF animals spontaneously elicited isoenergetic intake.

## Background

Obesity is characterized by the excessive accumulation of fat in the white adipose tissue (WAT) due to a chronic imbalance between energy intake and expenditure [1]. In fact, it is the surplus of energy derived from the metabolism of dietary carbohydrates, fats and proteins that end up stored as triglycerides (TGs) in adipocytes causing expansion of the WAT over time. In monogenic rodent models of obesity such as the *ob/ob *mouse and the *fa/fa *Zucker rat mutations in the leptin and the leptin receptor genes [2], respectively, lead to hyperphagia, defective non-shivering thermogenesis, and preferential deposition of energy in adipose tissue [3,4]. The metabolic alterations found in these animals clearly create a chronic positive energy balance condition that favors the development of obesity. However, since few cases of single gene mutations in obese human subjects have been identified [5], the physiological mechanisms underlying the excessive increase in adiposity in monogenic rodent models have to be interpreted with caution when applying to human obesity [6]. In fact, it is a combination of multiple genetic alterations that seems to predispose humans to obesity, although environmental and behavioral factors also influence the etiology of the obese phenotype [7]. Thus, in an attempt to generate an animal model of obesity that more closely resembles this disease in humans, fat-enriched diets or high-fat (HF) diets have been extensively used to induce obesity in rodents [6,8]. Indeed HF diet shows high efficacy in inducing obesity in mice and rats [6,8]; however, it is frequently reported that the excessive accumulation of adiposity caused by this approach in rodents is not necessarily accompanied by overfeeding [9-11]. In this context, we have recently found that male Wistar rats fed a HF diet for 8 weeks developed obesity [12], although the absolute and relative energy intake of these animals was not different from that of controls receiving standard rat chow. These findings are also in agreement with reports by others that obesity induced by HF diet in male Sprague Dawley rats was associated with modifications in the feeding pattern (higher meal size and reduced frequency of eating) rather than overfeeding, since the total number of calories ingested per day was not different between control and HF animals [11]. In another study, male Long-Evans rats fed a HF diet in amounts matching the energy intake of rats fed *ad libitum *a low-fat (LF) diet had significantly more adipose tissue than LF rats [9]. These findings indicate that besides energy density, the macronutrient composition of the diet plays an important role in determining the type and magnitude of adaptive metabolic responses elicited by the organism to feeding.

In cases where HF and standard chow-fed rats elicit isoenergetic intake, the much larger fat mass found in the former [9-12] has to derive from a condition of chronic energy surplus. In fact, based on the principle of energy conservation stated in the first law of thermodynamics, a systemic rearrangement of the mechanisms that regulate energy expenditure and substrate partitioning must occur in HF-fed rats in order to create a condition that exacerbates lipid storage in the WAT compartment. It appears that the organism prioritizes storage of energy in the WAT when chronically exposed to elevated dietary content of fat, an adaptive metabolic response that may occur independently of the total amount of energy ingested. From the perspective of prevention and treatment of obesity, it is particularly important to understand the physiological and molecular mechanisms by which extensive increases in adiposity occur in the absence of overfeeding. Of special interest are the adjustments that take place in whole-body energy balance and substrate partitioning in HF diet-induced obesity. Therefore, this study was designed to investigate the time-course of adjustments in several parameters that determine whole-body energy homeostasis in rats chronically exposed to a HF diet. Since metabolic alterations in brown and white adipose tissues are involved in adaptive thermogenesis [1,13,14], we have also assessed UCP-1 content in BAT and the ability of BAT and WAT to oxidize fatty acids in lean and obese animals. The content of acetyl-CoA carboxylase (ACC), a key enzyme in the *de novo *lipid synthesis pathway, was determined in the WAT and liver of control and HF rats after 8 weeks of dietary intervention. This study provides a detailed analysis of time-dependent whole-body and tissue-specific metabolic adaptations that underlie HF diet-induced obesity in rats. Interestingly, the macronutrient composition rather than the energy density of the diet was the main determinant of whole-body adaptive metabolic responses and increased adiposity in HF diet-induced obesity.

## Materials and methods

### Reagents

Fatty acid-free bovine serum albumin (BSA), palmitic acid, and phenylethylamine were purchased from Sigma-Aldrich. Human insulin (Humulin) was from Eli Lilly. [1-^14^C]palmitic acid was purchased from GE Healthcare Radiochemicals (Quebec City, Quebec, Canada). The uncoupling protein 1 (UCP-1) and GAPDH antibodies were from Abcam (Cambridge, MA). The acetyl-CoA carboxylase (ACC) antibody, β-actin antibody, and the secondary horseradish peroxidase (HRP)-conjugated anti-rabbit antibodies were from Cell Signaling Technology Inc. (Beverley, MA, USA). All other chemicals were of the highest grade available.

### Animals and diets

Male albino rats (Wistar strain) weighing 160 - 180 g were maintained on a 12/12 h light/dark cycle at 22°C and fed *ad libitum *either a low-fat rat chow (Control, 27.0%, 13.0%, and 60.0% of calories provided by protein, fat, and carbohydrates, respectively) with energy density of 3.43 kcal/g) or a high-fat (HF) diet (20%, 60.0%. and 20.0% of calories provided by protein [casein], fat [lard/soybean oil], and carbohydrates [maltodextrin/sucrose], respectively) with energy density of 5.16 kcal/g. All diets (standard diet cat # 5012 and high-fat diet cat # 58Y1) were purchased from TestDiet, Richmond Indiana (http://www.testdiet.com). All experimental procedures were approved by the York University Animal Care Ethics Committee.

### Determination of lean body mass (LBM)

LBM was determined by thoroughly dissecting the rats (12 controls and 12 HF) as previously described [12]. Briefly, after 8 weeks of either feeding a control or a HF diet, the rats were weighed, anesthetized (Ketamine/Xylazine 0.4 mg and 8 mg/100 g body weight), decapitated, and exsanguinated. A longitudinal anterior skin incision from neck to tail was then made. A scalpel was used to detach the entire skin consisting exclusively of fur and subcutaneous white adipose tissue (WAT) from the carcass of each animal. A similar procedure was carried out to remove the skin from the head. The head and body skins were weighed separately. The interscapular brown adipose tissue (iBAT) was removed, thoroughly trimmed of any visual WAT present. The abdominal and thoracic cavities were then longitudinally incised and the internal organs were exposed. The liver, kidneys, and heart, were carefully removed and individually weighed. The remaining abdominal organs as well as the lungs were all accounted for as viscera. Next, the retroperitoneal, epididymal, and inguinal fat depots were thoroughly removed and weighed. The mass of the carcass consisting of skeletal muscle and bones combined with the skinned head was used as LBM [12]. No significant differences were found with regards to the weights of liver, kidney, and heart between control and HF rats and these organs were not included in LBM.

### Determination of oxygen consumption (VO_2_), carbon dioxide production (VCO_2_), respiratory exchange ratio (RER), energy expenditure (heat production), and ambulatory activity

VO_2_, VCO_2_, RER, and energy expenditure were measured by indirect calorimetry using the Comprehensive Lab Animal Monitoring System (CLAMS) from Columbus Instruments. VO_2 _and VCO_2 _were normalized to the body weight of the animals and corrected according to an effective mass value [15]. RER is the ratio between the carbon dioxide production and the oxygen consumption (RER = VCO_2_/VO_2_). EE was calculated by multiplying the calorific value (CV = 3.815 + 1.232 **× **RER) by the observed VO_2 _(Heat = CV **× **VO_2_). The relative contribution of fat and glucose to the total heat produced was calculated by using RER and VO_2 _as previously described [15]. The CLAMS also performs 24 h-automated measurements of food intake and spontaneous physical activity. A system of infrared beams detect movement and ambulatory activity counts each time two successive beams are broken in the Z axis. Prior to the initiation of the HF feeding, two groups of rats (160-180 g) were randomly assigned to either the control or HF diet group. At this point, both groups of animals were receiving standard rat chow and were placed in the CLAMS for 24 h to record baseline values (week 0). Once the baseline values were collected, *ad libitum *feeding of the specific diets began and both groups of rats were placed in the CLAMS for 24 h once a week for 8 weeks (weeks 1 - 8). Prior to starting the recording of all variables, the animals were allowed to acclimatize in the metabolic cages for 1 h [12]. We have also kept rats in the CLAMS for up to 72 h and compared all metabolic parameters over 24, 48, and 72 h. We observed that the animals explore the new cage environment for about 15-20 min and then start reducing activity to the point that after 40-50 min of being in the cage environment most animals, if not all, are sleeping. This pattern of behavior was very consistent, since it repeated in all experiments we have carried out so far in the lab, including this study with animals fed a HF diet for 8 weeks. Additionally, all metabolic variables measured after the first hour of entering the CLAMS were similar at the respective time points after 24, 48, and 72 h in the cages. The acclimation data were excluded from the analyses. All experiments were carried out on Monday, with recordings starting at 1 pm and ending Tuesday at 2 pm. Two calorimeters were connected to the cages, which allowed the system to monitor all variables (VCO_2_, VO_2_, RER, heat, food intake, and activity) from 8 cages every 5 min, generating 12 data points/variable/animal/h.

### Determination of food intake, body mass, and energy efficiency

Body mass and food intake were monitored on a daily basis [12]. The diets were color-coded and allowed precise control of any spillage in the cages. Food intake was also monitored once a week by using the CLAMS and the values were identical to those obtained with manual determinations. The CLAMS provides a very precise assessment of food consumed by the animals. It contains individual balances that are installed underneath the feeding areas, allowing continuous monitoring of food consumed by each animal. The feeding area is designed in a way that prevents food from being mixed with urine or feces and eliminates spillage. It should be noted that with the feeding regimen adopted in this study all rats randomly assigned to the HF group ingested very similar amounts of food and also gained weight at a very similar rate throughout the study. Therefore, we could not clearly identify obesity-prone from obesity-resistant subgroups based on parameters such as weight gain as others have reported [16-18]. Furthermore, after 8 weeks of dietary intervention, all animals exposed to HF diet had a consistent and marked increase in subcutaneous and visceral fat, indicating that obesity was equally induced in all animals. Energy efficiency (EF) was calculated on a weekly basis by using the following equation: EF = weight gain (g/week/rat) ÷ energy intake (kcal/week/rat). Weight gain and energy intake were determined by subtracting body mass at the last day of the week from the corresponding value of the previous week and by multiplying food intake by the energy density of the diet for each specific week, respectively.

### Determination of circulating leptin

Blood (~350 μl) was collected in heparin-coated tubes once a week from the saphenous veins of control and HF animals [12]. Collection occurred every Wednesday at 11:00 am with animals under fed conditions. Blood samples were centrifuged and the plasma was stored at -80°C for further analysis. Leptin was measured by ELISA using a commercially available kit.

### Measurement of palmitate oxidation in BAT homogenates and isolated epididymal adipocytes

For the preparation of homogenates, ~100 mg of (BAT) were thoroughly minced in 200 μL of ice-cold homogenization buffer (SETH buffer = 250 mmol/l sucrose, 1 mmol/l EDTA, and 10 mmol/l Tris-HCl, pH 7.4). Additional SETH buffer was added to yield a 20-fold (wt/vol) diluted minced tissue sample. The solution was then homogenized in an ice-cold Potter-Elvehjen glass homogenizer (10 passes across ~30 seconds). Subsequently, 400 μl of BAT homogenate were transferred to a plastic scintillation vial containing 1.6 ml of the reaction mixture (100 mmol/l sucrose, 80 mmol/l KCl, 10 mmol/l Tris-HCl, 5 mmol/l KH_2_PO_4_, 1 mmol MgCl_2_, 2 mmol/l malate, 2 mmol/l ATP, 1 mmol/l dithiothreitol, and 0.2 mmol/l EDTA, 2 mmol/l L-carnitine, 0.05 mmol/l CoA, 0.2 μCi/ml [1-^14^C]palmitic acid, and cold palmitate (0.2 mM) complexed with fat-free BSA). Palmitate oxidation by BAT was measured by the production of ^14^CO_2 _from [1-^14^C]palmitic acid (0.2 μCi/ml) with nonlabeled palmitate (200 μM) present in the medium as described previously [19]. The flasks where tissue homogenates were incubated had a centered isolated well containing a loosely folded piece of filter paper moistened with 0.2 ml of 2-phenylethylamine/methanol (1:1, v/v). After the 1 h-incubation period, the media were acidified with 0.2 ml of H_2_SO_4 _(5N), and the flasks were maintained sealed at 37°C for an additional 1 h for collection of the ^14^CO_2 _released. Subsequently, the filter papers were carefully removed and transferred to scintillation vials for radioactivity counting [19]. Palmitate oxidation by isolated epididymal adipocytes was measured by the production of ^14^CO_2 _from [1-^14^C]palmitic acid (0.2 μCi/ml) with nonlabeled palmitate (200 μM) present in the medium as described previously [19]. The flasks where either tissue homogenates or isolated adipocytes were incubated had a centered isolated well containing a loosely folded piece of filter paper moistened with 0.2 ml of 2-phenylethylamine/methanol (1:1, v/v). After the 1 h-incubation period, the media were acidified with 0.2 ml of H_2_SO_4 _(5N), and the flasks were maintained sealed at 37°C for an additional 1 h for collection of the ^14^CO_2 _released. Subsequently, the filter papers were carefully removed and transferred to scintillation vials for radioactivity counting [20,21].

### Western blot determination of UCP-1, ACC, GAPDH contents in BAT, epididymal fat, and liver

Immediately after extraction, BAT, epididymal fat, and liver samples were snap-frozen in liquid nitrogen and homogenized in buffer containing 25 mM Tris-HCl, 25 mM NaCl, 1 mM MgCl_2_, 2.7 mM KCl, 1% Triton-X, 10% glycerol, and protease and phosphatase inhibitors (0.5 mM Na_3_VO_4_, 1 mM NaF, 1 μM leupeptin, 1 μM pepstatin, and 1 mM PMSF) [19-21]. After protein determination in each sample, aliquots of tissue lysates were then subjected to SDS-PAGE, transferred to PVDF membranes, and then blotted for ACC, UCP-1, and GAPDH.

### Statistical Analyses

Statistical analyses were performed by using two-way ANOVA with Tukey-Kramer multiple comparison post-hoc tests or t-tests as indicated in the figure legends. The level of significance was set to P < 0.05.

## Results

### Body mass, LBM, food intake, energy intake and efficiency, and adipose tissue and muscle masses

**Figure 1:**
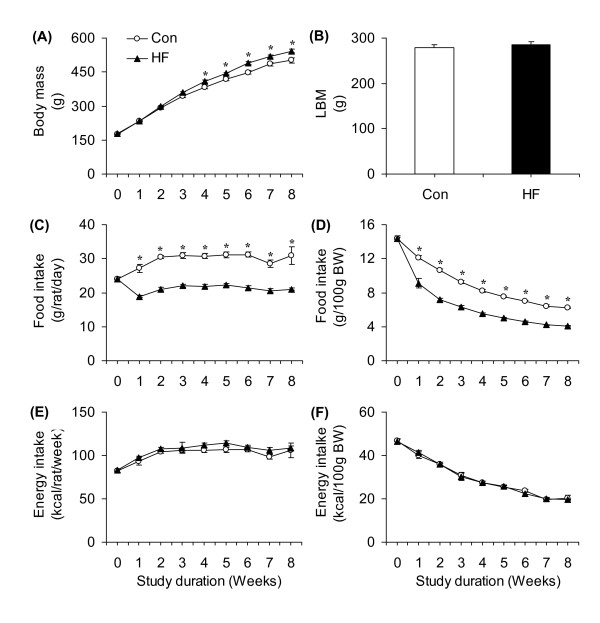
**Time-course profile of body mass (**A**), lean body mass (LBM, **B**), absolute (**C**) and relative (**D**) food intake, as well as absolute and relative (**E**) to body weight (**F**) energy intake of rats either fed standard chow (Control, Con) or a high-fat (HF) diet for 8 weeks**. *P < 0.05 vs. Con. N = 20.

**Figure 2:**
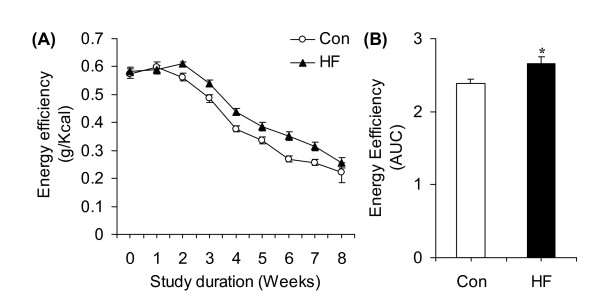
**Time-course profile of energy efficiency (**A**) of rats either fed standard chow (Control, Con) or a high-fat (HF) diet for 8 weeks**. Area under the curve (AUC, **B**) for energy efficiency *P < 0.05 vs. Con. N = 20.

**Figure 3:**
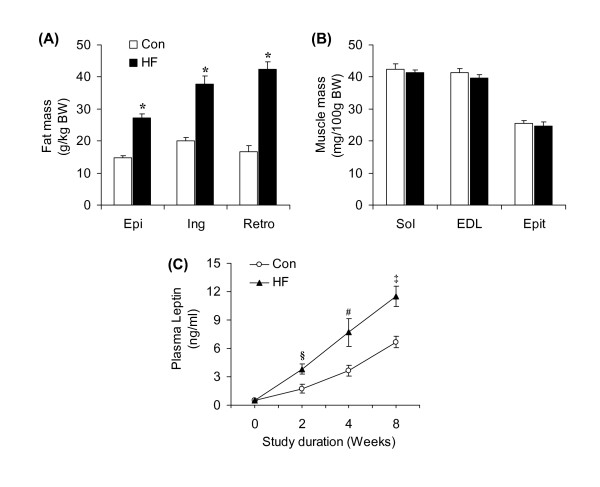
**Effects of either a standard (Control, Con) or a high-fat (HF) diet on epididymal (Epi), inguinal (Ing), and retroperitoneal (Retro) fat pad masses **(A)**, as well as on soleus (Sol), extensor digitorium longus (EDL), and epitrochlearis (Epit) muscle masses **(B)**, N = 12**. Time-course analysis of plasma leptin **(C) **and adiponectin **(D) **throughout the 8-week dietary-intervention period, N = 8. *P < 0.05 vs. respective Con. ^§ ^P < 0.05 vs. Con week 2 and all other HF conditions. ^#^P < 0.05 vs. Con week 4 and all other HF conditions. ^‡^P < 0.05 vs. Con week 8 and all other HF conditions. N = 8.

Body mass was similar in both groups at week 0 (~177 g), week 1 (~232 g), and week 2 (~294 g). However, body mass started to diverge between the two groups at week 3 and this variable was higher in HF animals (~8%) than in controls at the week 8 (Figure 1A). LBM did not differ between control and HF rats at the end of the study (Figure 1B). Food intake in grams per rat per day was similar for both groups at baseline (week 0) then reduced in the HF diet group by ~30% compared to controls (Figure 1C). Analysis of food intake relative to body mass also revealed that this variable was consistently reduced by ~25 - 31% at week 1 and by ~30 - 35% from weeks 2 to 8 (Figure 1D). Interestingly, HF rats spontaneously adjusted food consumption such that energy intake either in absolute values (Figure 1E) or corrected for body mass (Figure 1E) was similar in both groups of animals throughout the entire 8-week period. Energy efficiency did not differ between control and HF rats in week 0 and week 1 of the study. However, this variable increased by ~8.5, 11.0, 16.0, 14.0, 31.0, 23.0, and 16% at weeks 2, 3, 4, 5, 6, 7, and 8 of the study, respectively (Figure 2A). The area under the curve for energy efficiency from week 2 to 8 was ~12% higher in HF than control animals (Figure 2B). Despite spontaneous isoenergetic intake in animals fed *ad libitum*, at the end of the 8-week-period, the epididymal, inguinal, and retroperitoneal fat masses were ~1.85-, ~1.9-, and ~2.5-fold higher in the HF-fed than in the regular chow-fed rats (Figure 3A). BAT mass was also increased by 1.5-fold in HF compared to controls (Figure 3B), while the masses of soleus, EDL, and epitrochlearis muscles did not differ between control and HF rats (Figure 3C).

### Leptin

Plasma leptin was similar in both groups at the beginning of the study (~0.55 ng/ml). However, after 2, 4, and 8 weeks of dietary intervention, leptin levels were ~2.2-, 2.1-, and ~1.73-fold higher in HF rats (3.82, 7.69, and 11.52 ng/ml, respectively) than controls (1.74, 3.64, and 6.67 ng/ml, respectively) (Figure 3D).

### O_2 _consumption, CO_2 _production, RER, and energy expenditure

**Figure 4:**
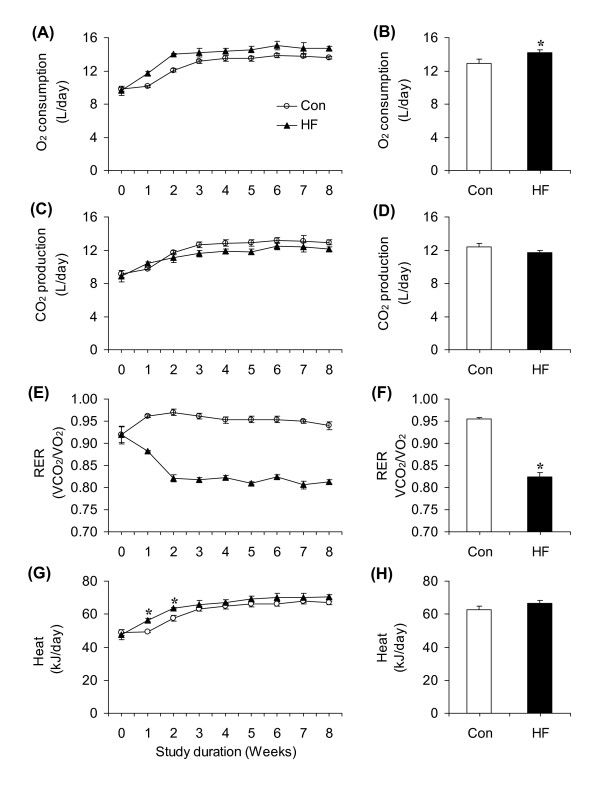
**Time-course adaptations of O_2 _consumption (**A**), CO_2 _production (**C**), RER (**E**), and heat (**G**) in rats either fed a standard chow (control, Con) or a high-fat (HF) diet for 8 weeks**. Bar graphs represent average values for O_2 _consumption (**B**), CO_2 _production (**D**), RER (**F**), and heat production (**H**) over the 8-week dietary intervention period. *P < 0.05 versus Con. N = 8 - 12. *P < 0.05 vs. Con.

Baseline values for O_2 _consumption did not differ (~9.7 L/day) between the two groups (Figure 4A). However, this variable was consistently higher in HF than controls throughout the study, with the highest increases in O_2 _consumption occurring at weeks 1 (15.8%) and 2 (16.1%). From week 2 on, the differences in O_2 _consumption between control and HF animals reduced to ~8% and remained at that level until the end of the study (Figure 4A). The average increase in O_2 _consumption from week 1 to 8 for the HF groups was ~9.6% (12.94 and 14.18 L/day for the control and HF rats, respectively) (Figure 4B). In the first 2 weeks of the study, CO_2 _production did not differ between control and HF animals. However, from week 3 to 8, CO_2 _production remained consistently lower in HF than controls (Figure 4C). The average CO_2 _production from week 1 to 8 did not differ between control and HF rats (12.37 and 11.71 L/day, respectively) (Figure 4D). Baseline values for RER were similar in both groups (~0.919), and then reduced to 0.883 at week 1 and to 0.824 at week 2. From week 3 to 8, RER remained at a significantly lower level in HF than controls (Figure 4E). The average RERs from week 1 to 8 for the control and HF rats were 0.955 and 0.824, respectively (Figure 4F). Baseline heat production was similar in both groups (Figure 3G) and then significantly increased by ~14% at week 1 and by ~11% at week 2 in HF rats. From week 3 to 8, no differences in heat production were detected between the two groups (Figure 4G and 4H).

### Ambulatory activity during the light and dark cycles

**Figure 5:**
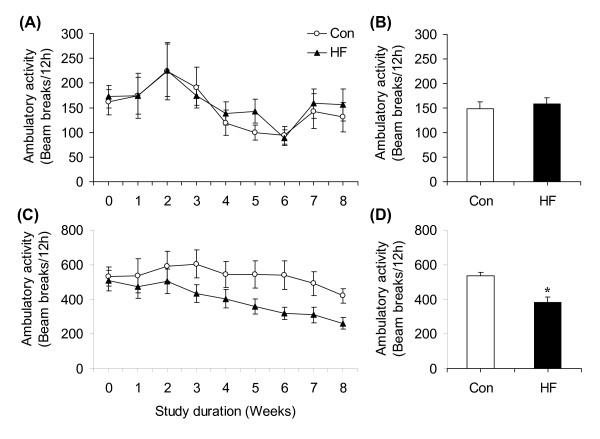
**Time-course profile of average light cycle **(A) **and dark cycle **(C) **daily ambulatory activity of rats either fed standard chow (control, Con) or high-fat (HF) diet for 8 weeks**. Bar graphs represent average ambulatory activity over the 8-week dietary-intervention-period during the light **(B) **and dark **(D) **cycles. *P < 0.05 vs. Con. N = 12.

Ambulatory activity did not differ between control and HF rats (Figure 5A and 5B) during the light cycle. However, HF animals elicited a progressive reduction in this variable during the dark cycle, which became evident after week 2 (Figure 5C). In fact, dark cycle ambulatory activity for HF rats was consistently reduced by ~29, 26, 35, 40, 37, and 38% at weeks 3, 4, 5, 6, 7, and 8, respectively, compared to control rats (Figure 5C). The average reduction in ambulatory activity of the HF animals during the dark cycle throughout the 8-week-period was ~28.3% (Figure 5D).

### UCP-1 content, BAT mass, BAT and WAT palmitate oxidation, and ACC content in WAT and liver

**Figure 6:**
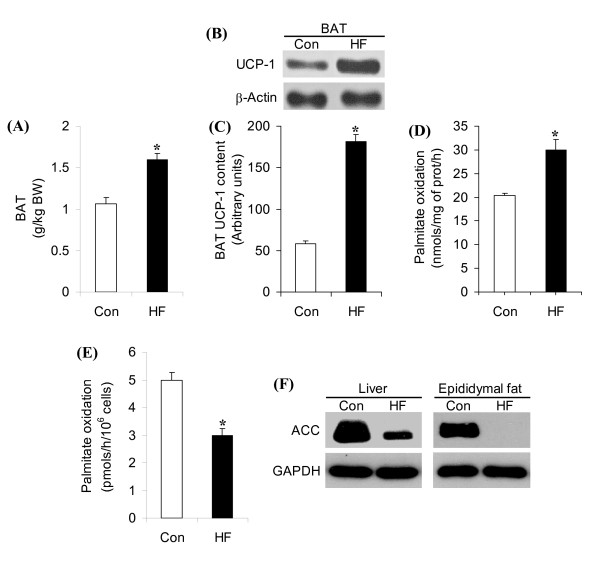
**Effects of high-fat (HF) diet on brown adipose tissue (BAT) mass (**A**), BAT uncoupling protein-1 (UCP-1) content (B and C), palmitate oxidation in BAT (D) and in isolated epididymal adipocytes (E), and the content of acetyl-CoA carboxylase (ACC) in liver and epididymal fat (F)**. Representative blots for UCP-1 in BAT (B) and for ACC in liver and epididymal fat (F) of control (Con) and HF rats after 8 weeks of dietary intervention. β-actin and GAPDH were used as loading controls. *P < 0.05 vs. Con. N = 12.

BAT mass increased by 1.5-fold (Figure 6A) in rats fed a HF diet for 8 weeks when compared to controls. The content of UCP-1 in BAT of HF rats was 3.1-fold higher than controls (Figure 6B and 6C). Analysis of palmitate oxidation in BAT homogenates and in isolated epididymal white adipocytes revealed that this variable increased by 1.47-fold (Figure 6D) in the former and reduced by ~40% in the latter (Figure 6E) when compared to control. ACC content in epididymal fat depot and liver was markedly reduced in these tissues after 8 weeks of HF diet (Figure 6F).

## Discussion

Here, we demonstrate that despite spontaneous isoenergetic intake, rats fed *ad libitum *a HF diet accumulated substantially more visceral and subcutaneous fat than rats fed standard chow. In fact, within 3 to 4 days on the HF diet, food consumption was adjusted to precisely match the energy intake elicited by control rats. This was observed either when food intake was assessed relative to body weight or in amounts per animal, indicating that alterations in the energy density of the diet were rapidly detected and food intake was self-regulated accordingly throughout the entire duration of the study. It was remarkable that the masses of visceral (epididymal and retroperitoneal) and subcutaneous (inguinal) fat pads were ~1.85- to 2.5-fold higher in HF rats than controls, even though energy intake was similar between both groups of animals and body mass in HF rats was only ~8% higher than controls. In fact, the weight gained was strictly towards fat accumulation, since no differences in LBM between control and HF rats were found after 8 weeks of dietary intervention. This is in line with previous observations that rats fed a HF diet for 10 weeks displayed only a 10% increase in total body weight, but fat pads weighed 30-45% more than those of animals fed a low-fat diet [9]. This is also compatible with the fact that the density of fat is significantly lower than other components of fat-free mass [22], which reduces the impact of increased adiposity on total body mass. We also found that 24 h O_2 _consumption was ~16% higher in HF than controls in weeks 1 and 2, and then reduced to ~8% from weeks 3 to 8. Whole-body energy expenditure was also higher in HF rats than controls at weeks 1 and 2, a difference that no longer existed after week 3, indicating that a transient increase in energy expenditure occurred in HF rats. Interestingly, RER was reduced within the first two weeks of the animals being on the HF diet, demonstrating that whole-body substrate metabolism was progressively shifted towards fat oxidation. Fat actually supplied ~25%, 39.6%, and 60.5% of the energy expended at baseline, week 1, and 2 of HF feeding, respectively. From week 3 to week 8, RER remained relatively constant. Therefore, although food intake was adjusted within 3 to 4 days, it took ~2 weeks for whole-body substrate partitioning to be fully adjusted once the animals were placed on a HF diet.

We had previously demonstrated that the dark-cycle ambulatory activity was significantly reduced in rats exposed for 8 weeks to HF diet [12]. However, it was not clear at what point during the course of HF feeding this adaptation occurred. An early reduction in spontaneous physical activity in HF animals could restrain energy expenditure and contribute to obesity development. In fact, we found a progressive reduction in dark-cycle ambulatory activity in HF rats; reaching values ~38% lower than controls at week 8. The onset of this reduction occurred at week 2 of HF diet when adjustments in food intake and whole-body substrate partitioning had already occurred. These findings are consistent with sensing energy availability and triggering alterations in peripheral metabolism that regulate energy expenditure accordingly [14,23,24]. However, the progressive reduction in dark-cycle ambulatory activity in rats fed a HF diet seems counterintuitive at first, since energy expenditure would be expected to increase in an attempt to maintain body mass relatively constant over time. Although our data demonstrated that energy expenditure of HF rats was indeed higher than controls in Weeks 1 and 2 of the study, this was not sustained thereafter. Importantly, equalization of energy expenditure between the two groups coincided with the time of reduction in ambulatory activity in HF rats. This indicates that the initially increased thermogenic response in HF rats was counteracted by a reduction in dark cycle spontaneous physical activity, which decreased energy expenditure and facilitated adipose tissue expansion in these animals.

This study assessed several components of the complex system that regulates whole-body energy homeostasis and revealed that besides energy density, the nutrient composition of the diet played a major role in determining whether whole-body energy expenditure was increased or reduced. The mechanisms underlying these adaptive metabolic responses are still unclear. Previous studies have suggested that HF diet-induced obesity is associated with increased calories per meal rather than per day [11]. Time and rhythmicity of feeding have indeed been associated with obesity in rodents and humans [8]. However, it still does not explain the origin of the energy surplus required for HF rats to have markedly higher than control adiposity [9-12], since both groups of animals elicited isoenergetic daily intake. Importantly, in this study and those of others [9-11] energy intake was assessed based on the amount of food consumed without precisely determining nutrient absorption by the gastrointestinal (GI) tract. It is possible that alterations in the nutrient composition of the diet could alter the gut microbiota toward more efficient extraction of energy from the diet and lead to obesity. In fact, a gut microbiome with increased capacity for energy harvest has been associated with obesity in humans [25]. Moreover, recent studies have provided evidence that switching humanized gnotobiotic mice from a low-fat-plant poly-saccharide rich-diet to a high-fat/high-sugar Western diet quickly (within one day) shifted the structure of the gut microbiota and altered microbiome gene expression [26], indicating that the macronutrient composition of the diet may drastically affect GI tract function. Therefore, further studies are warranted to investigate whether feeding a HF diet alters the gut microbiota in a way that increases nutrient absorbance by the GI tract and facilitates the development of obesity in HF diet-induced obesity.

An alternative possible explanation for our intriguing findings and of others [9-11] is that the high availability of fat disrupted the normal operation of the system that senses and regulates adipose tissue metabolism and whole-body energy expenditure. In fact, in order for the brain to regulate non-exercise activity thermogenesis (NEAT) according to changes in energy balance, the hypothalamus and other brain regions need to integrate external sensory cues of energy availability with internal endocrine and metabolic signals arising from various organs and tissues [27]. In this scenario, the adipose-derived hormone leptin plays a major role communicating to the hypothalamus the amount of energy stored in the organism [23,27,28]. As fat mass increases so does the expression and secretion of leptin by adipocytes leading to a central nervous system (CNS)-mediated reduction in food intake and up-regulation of NEAT [23,27,28]. In this study, we found that circulating leptin was significantly higher in HF than control rats after just 2 weeks of the dietary intervention. However, we have recently reported that the protein content of the suppressor of cytokine signaling 3 (SOCS3), a marker of leptin resistance, was increased in the hypothalami of HF rats [12]. Also, while hypothalamic AMPK activation is expected to be downregulated by increased leptin [29], we found that this variable was actually higher in HF than control rats [12], indicating hypothalamic leptin resistance in our HF rats. Therefore, the reduction in ambulatory activity and the inability to sustain the initial increase in energy expenditure in HF rats could, at least partially, be due to impaired leptin signaling in the CNS of these animals. These centrally-mediated effects must also have increased the ability of the adipose tissue to store fat as demonstrated by the markedly increased adiposity in rats chronically fed a HF diet. Interestingly, major lipogenic organs such as liver and adipose tissue in HF rats markedly reduced their ACC content, which indicates that the *de novo *lipid synthesis pathway was potently suppressed in these animals. These findings are compatible with the fact that there was no need to generate long-chain fatty acids (LCFAs) in a condition where the diet already supplied large amounts of this substrate. Additionally, suppression of the *de novo *lipid synthesis pathway must have increased the efficiency of nutrient storage in adipose tissue, since there is a high energy cost associated with building LCFAs from glucose or aminoacids [8].

In order to assess whether BAT contributed to the shift in whole-body fat oxidation and to the thermogenic response to HF diet, we measured UCP-1 content and palmitate oxidation in this tissue. The increase in UCP-1 content and palmitate oxidation in BAT shows that an adaptive thermogenic response was induced by HF diet. However, it appears that the reduction in NEAT and activation of other tissue-specific metabolic adjustments offset the impact of BAT-induced thermogenesis on whole-body energy expenditure in HF rats. In this context, we found that fat oxidation in the adipocytes isolated from the epididymal fat pad was reduced by 40% in HF rats, which is in line with our previous study showing that fatty acid oxidation was potently reduced in visceral and subcutaneous adipocytes from mice exposed to HF diet for 8 weeks [20]. Although there is no evidence that FA oxidation in adipocytes increases as a means to cope with excess lipid load in obesity, our data suggest that the impairment of FA oxidation in WAT may further contribute to the accumulation of fat mass in both visceral and subcutaneous fat depots under conditions of HF-diet-induced obesity.

In conclusion, *ad libitum *HF feeding induced time-dependent adaptive responses in food consumption that maintained energy intake at the same level of standard-chow-fed animals. Increased whole-body fat oxidation and UCP-1 content in BAT of HF rats were counteracted by the reduction in spontaneous physical activity during the dark cycle in these animals. The largely expanded adipose tissue of HF rats was very efficient in storing lipids and displayed significantly reduced fat acid oxidation. Also, a marked reduction in the content ACC in fat tissue and liver indicated that the costly process of *de novo *lipid synthesis was potently suppressed. Interestingly, the increased ability of the adipose tissue to store large amounts of energy under conditions of high dietary fat availability occurred in the absence of overfeeding, indicating that these adaptive responses were mainly driven by the macronutrient composition of the diet. These findings provide novel information regarding time-dependent adaptations to HF diet that may be of relevance for understanding the physiopathology of diet-induced obesity.

## Competing interests

The authors declare that they have no competing interests.

## Authors' contributions

MS and BM carried out all experiments using the CLAMS, performed serial blood collections, measured all blood parameters, and organized the data. MPG measured fatty acid oxidation in liver and isolated adipocytes and carried out western blots for UCP-1 and ACC. RBC designed the experiments, helped conducting experiments, and wrote the manuscript. All authors read and approved the manuscript.
